# External root thermal analysis of three different obturation techniques 

**DOI:** 10.4317/jced.61035

**Published:** 2024-01-01

**Authors:** José-Cordeiro-Lima Neto, Fernanda-Clotilde-Mariz Suassuna, Diego-Filipe-Bezerra Silva, Ramon-Targino Firmino, Patrícia-Meira Bento, Daniela-Pita de Melo

**Affiliations:** 1 Department of Oral Diagnosis, Division of Oral Radiology, State University of Paraíba- UEPB, Campina Grande, Paraíba, Brazil; 2 Faculty of Nursing and Medicine Nova Esperança, João Pessoa, Paraíba, Brazil; 3 Academic Unit of Biological Sciences, Federal University of Campina Grande – UFCG, Patos, Paraíba, Brazil; 4 College of Dentistry, University of Saskatchewan, Saskatchewan, Canada

## Abstract

**Background:**

The purpose of this study was to assess the external root surface thermal behavior when submitted to three different obturation techniques.

**Material and Methods:**

Forty-five single-rooted premolars were selected, prepared and randomly divided into three groups according to the studied obturation techniques: lateral condensation (LC), single cone technique (SCT) and injectable thermoplasticized technique (IT). Each tooth was placed in a customized apparatus and connected to a thermocouple. A FLIR T650sc infrared thermal camera was used to assess root temperature in a room under controlled temperature and humidity. Temperature values were recorded using the thermal camera and thermocouples before, during and 30, 60, 90, 120, 150s after obturation. Shapiro-Wilk, QQ-plot, Levene’s, ANOVA-three-way, Mauchly’s sphericity, Box’s M and Bonferroni tests were used to assess data. The significance level was set at 5%.

**Results:**

Infrared assessment showed significant temperature changes between time intervals, obturation techniques and root thirds. Temperature increase was observed 30s after obturation for STL, LC and IT, followed by a gradual temperature decline, with temperature values similar to the initial temperature at 150s for LC and IT. SCT temperature values only returned to normal 60s after obturation in the apical third and 90s in the middle and cervical thirds. In all techniques, the temperature did not rise above the critical limit of 10°C.

**Conclusions:**

All studied obturation techniques increased root surface temperature with IT showing the highest temperature increase. However, the temperature increase does not exceed the acceptable limits, not causing damage to the surrounding tissues.

** Key words:**Endodontics, changes in body temperature, root canal filling, thermography.

## Introduction

Obturation of the root canal system is an important step towards root canal treatment success. After cleaning, disinfection and shaping, obturation of the entire root canal system is required using suitable filling materials to prevent reinfection. Usually, a combination of gutta-percha as the core material and an endodontic sealing agent is used to fill the root canals (1). The quality of the root canal filling is evaluated according to two main technical variables: its length in relation to the radiographic apex and its density, indicating the absence of voids within the filling material (2). To adapt the filling material to anatomical variations, different techniques have been proposed, all presenting satisfactory performance repairing endodontically treated teeth when well performed (3,4). Therefore, the endodontist’s concern lies on postoperative comfort and long-term preservation of the tooth and its supporting structures.

Warm endodontic filling techniques thermoplasticize gutta-percha or use heated compactors to an average of 200°C to plasticize gutta-percha. The temperature rise caused by some of these filling techniques is radiated to the external surface of the root, even though the dentin has low thermal conductivity (5). The periodontal ligament and other periodontal tissues may have their health temporarily or permanently impaired by the rise in temperature (5). The rise in dentin temperature 10°C above the body temperature also initiates changes in regional microcirculation, generating damage to the adjacent connective tissue, which can lead to problems such as chronic periodontitis and tooth resorption. Such damage may be irreversible if the temperature exceeds 16°C above the body temperature (6).

The increase in temperature due to the use of heat may also influence the characteristics of the filling materials, with consequences to the success of the endodontic treatment. Previous studies have assessed heat increase interference on cement compatibility and on its physical (viscosity, setting time, flow) and chemical properties (7-10).

In order to assess temperature, increase in different obturation techniques, thermography appears as a non-ionizing and non-invasive method, which captures and records the thermal distribution with the help of thermal cameras that can detect infrared radiation emitted by objects. The thermal map image is called thermogram, which is generated in real time, enabling the measurement of temperatures and the observation of patterns of heat distribution. Thermograms can be acquired with high resolution on modern infrared thermal cameras, allowing realistic measurements of the area of interest when reflections are avoided and the temperature is kept constant (11,12).

As a complementary analysis, thermocouples are sensors that can measure temperature due to the “Seebeck Effect”, which occurs when two wires of different metals are joined at both ends and one of them is heated, generating a flow of direct current in the thermoelectric circuit. This sensor must be connected to a thermometer or other device capable of recording data. Thermocouples are known for their versatility and are used on dentistry (13).

A deeper understanding of the interference and consequences of temperature increase will enable clinicians to better understand the different filling techniques, combining practicality, predictability, and safety, resulting in important clinical gains without causing damage to the supporting tissues. Therefore, the aim of this study was to evaluate the variation in the thermal behavior of the external root surface when submitted to three different endodontic filling techniques, observing the temperature variation of these roots after the procedure.

## Material and Methods

-Sample selection

Forty-five single-rooted premolars with complete root development with single root canals and ≤ 5° of angulation were selected. All teeth were cleaned and disinfected, and then stored in 0.9% NaCl saline solution. The sample was then radiographed with digital sensor for periapical radiographs (Acteon – Microimagem, Indaiatuba, SP, Brazil). Teeth that presented pulp nodules, internal resorption, previous endodontic treatments, or root fracture were excluded from the sample.

-Sample Preparation

The coronal portion of all teeth were removed perpendicular to the long axis of the root, at the level of the cementoenamel junction with the aid of a diamond disk and then separately inserted into polypropylene tubes with a 0.9% NaCl saline solution. The teeth and tubes were numerically identified in terms of working length (WL), instrument size and obturation technique used. All teeth selected were prepared using the same memory instrument, to keep a standardized sample.

The WL was established as actual tooth length subtracted 1 millimeter (mm). In order to fix the sensitive ends of the thermocouples, spherical grinding was performed on the lingual surfaces with a diamond bur and the points were measured and marked equidistantly on the three thirds of the teeth with a depth of approximately 1 mm.

-Root canal instrumentation

The root canal was irrigated with 2 ml of 2.5% sodium hypochlorite using an irrigation syringe and endo-eze needle (Mk Life, Porto Alegre, RS, Brazil). Number 10 K-type hand files were introduced up to the apical foramen and the WL was determined. For root canal preparation, a NiTi Reciproc (VDW GmbH, Munique, Germany) file was used, according to the manufacturer’s recommendations.

As for the kinematics of movement, the indication of passive use was followed, using pecking movements, with slow advances until reaching the real working length. After reaching the desired length, brushing movements were made on all the walls of the root canal to remove all the content present, always with constant irrigation with 5 ml of 2.5% sodium hypochlorite.

After preparation, the root canals underwent a final irrigation with 2ml 17% EDTA for 3 minutes, followed by a new irrigation with 5ml of sodium hypochlorite.

-Thermal image acquisition

Thermograms were acquired using a portable infrared sensor camera FLIR Model T650sc (FLIR, Wilsonville, Oregon, USA), with a 25 mm lens and spatial resolution of 640 x 480 pixels. The camera was fixed on a tripod to perform the images in a controlled environment with standardized temperature. A digital thermohygrometer was used to monitor the temperature and relative humidity of the air at 16°C and 40 to 60%, respectively.

To avoid thermal interference, an apparatus to hold and isolate the sample was built, consisting of a thermal box of expanded polystyrene plates, with its internal walls coated with a layer of aluminum foil covered by black Ethylene Vinyl Acetate (EVA). To delimit the field of view, a rectangular opening was made at the point where the images should be captured.

The thermal camera was positioned with a distance of 30 cm from the sample to maintain high resolution and precision for the selected lens following the manufacture’s indications. The following parameters were set in the thermal camera: distance 30 cm, corresponding to the smallest possible distance allowed by the lenses; emissivity of 0.98 stipulated for human tissues and relative humidity of 44%.

To register the temperature changes in real time, videos were made during the procedures (obturation techniques) of the infrared camera and thermocouples. Three points corresponding to the drills made to position the thermocouples were used to register the temperature measurements from the infrared camera. The initial temperature, the highest temperatures achieved, the difference between acquired temperatures and temperatures at different time intervals after obturation (30, 60, 90, 120 and 150 seconds) were collected for each assessed tooth.

-Thermal analysis using a thermocouple

A four-channel digital thermometer (RDXL4SD, Omega Engineering, USA) with three K-type thermocouples was used, with a resolution of 0.1°C in the temperature range of -50.0 to 999.9°C.

The thermocouples were positioned in a rectangular thermal insulated polystyrene with three holes equidistant from each other and directed to the drills made on each tooth. A thermocouple electrode was inserted into each hole and connected to the thirds of the teeth. The thermometer attached to the thermocouples was positioned externally to the insulated polystyrene box and filmed using an external camera. All temperature values were registered for posterior data analysis.

-Root Canal Obturation Techniques

After instrumentation of the root canal, the teeth were divided into three groups and submitted to the obturation protocols. The sample was equally divided into three groups according to the obturation technique used: 1- Lateral condensation, 2- Single cone technique and 3 - Injectable thermoplasticized gutta-percha technique. All protocols used absorbent paper cones from the reciprocating system to dry the root canal and AH plus resin cement (Dentsply Maillefer, Ballaigues, Switzerland).

For the lateral condensation technique, gutta-percha cones using ISO (02) taper were used. The main cone was selected according to the size of the last instrument used during root preparation. Thus, taper cones 25.02 were selected according to the instrumentation and measured according to the working length of the tooth. The accessory cones were selected according to the spacer and the space to be filled.

After drying the canal, cement was applied on the cones and canal walls. A NiTi spacer (Dentsply Maillefer, Ballaigues, Switzerland) was used to provide adequate space for the insertion of accessory cones. This process was repeated until the cervical portion was filled. The excess of gutta-percha in the cervical region was removed with heat and the coronal portion was compacted.

The single cone technique was performed by adapting a cone of size and conicity identical to the last instrument used in the mechanical preparation. After drying the canal, the cement was brushed on the walls of the conduit with the cone itself, and then inserted into the actual working length. The gutta percha cone was removed with heat and the plastic mass was vertically compacted.

For the injectable thermoplasticized gutta-percha technique, cement was applied with the aid of an ISO series cone based on the last instrument used during preparation. Afterwards, the gutta percha stick was placed in the thermoplasticizing injector device (Mk Life, Porto Alegre, RS, Brazil) and it was automatically heated until 220 degrees Celsius, according to the manufacturer’s instructions. When the device signaled that it was ready to start obturation, the plastic filling material filled the canal in thirds incrementally, from the apical to the cervical third. After the canal filling was completed, the plastic mass was vertically compacted.

-Statistical analysis

Data were statistically analyzed using the Statistical Package for Social Sciences program (SPSS, v. 25.0, IBM, Chicago, IL, USA) for Windows. A Gaussian distribution was tested using the Shapiro-Wilk test and a QQ-plot analysis. Levene’s test was used to prove the homogeneity of variance and the temperature evaluated, both by thermocouple and infrared thermographic analysis, were the dependent variables. The data followed a Gaussian distribution and a homogeneity of variance. Exploratory statistics consisted of mean and standard deviation. A general linear model / three-way mixed model analysis of variance (ANOVA) was used to test the intra-subjects’ factor (time: temperature over time) and two between-subjects’ factors (Filling Technique and Anatomical Region) and the interaction between these factors. The results of Mauchly’s sphericity test and Box’s M test were used to evaluate the ANOVA assumptions: sphericity and assumption of homogeneity of multiple variance-covariance matrices, respectively. The Mauchly sphericity violation (*p*<0.05) was corrected using the Greenhouse-Geisser correction due to the Greenhouse-Geisser epsilon (less than 0.75, for both analyses). In addition, the main interaction effects were tested for group and time comparisons. Bonferroni adjustment was applied for multiple comparisons.

## Results

-Temperature assessed with Thermocouples

**Figure 1:**
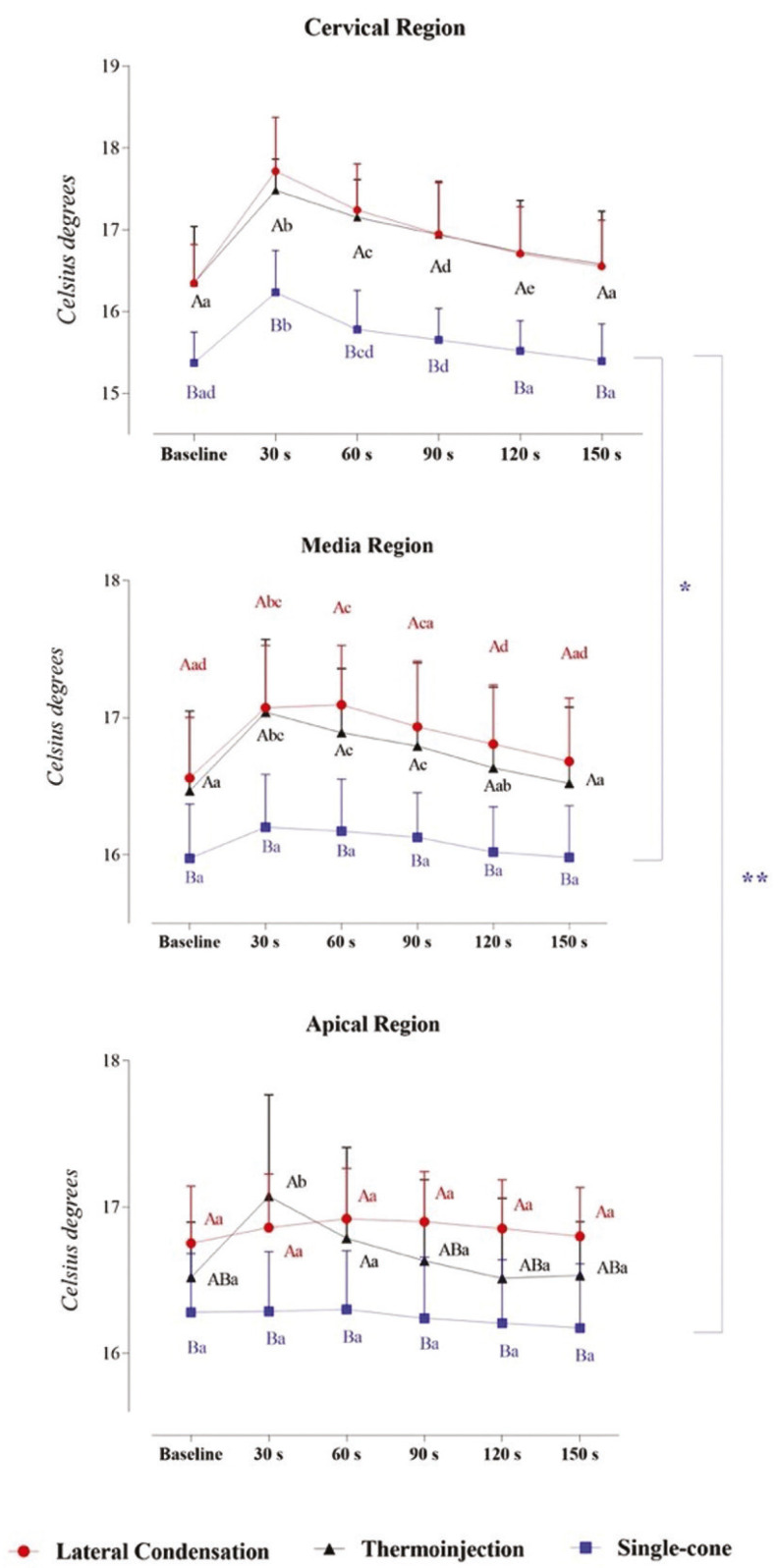
Interaction between time, filling technique, and third in thermocouple analysis: a Three-way Mixed Model. Statistical analyses were performed with a sample of 45 cases, 15 per group. ηp2: Partial eta squared. Data plotted as mean and standard deviation. Supplementary material provides complementary information regarding data statistics. To overcome sphericity violation, the Greenhouse-Geisser correction were applied. Main effects were suppressed due to significant interactions. Interaction effects for Time and Filling technique: α = 0.001, β-1 = 0.963, ηp2 = 0.058). Interaction effects for Time and Anatomic region: α = 0.000, β-1 = 1.000, ηp2 = 0.228. Interaction effects for Filling technique and Anatomic region: α = 0.004, β-1 = 0.898, ηp2 = 0.112.

Figure 1 shows the three-way mixed model ANOVA and Bonferroni test for multiple comparisons. Table 1 provides complementary information regarding data statistics.

**Table 1:**
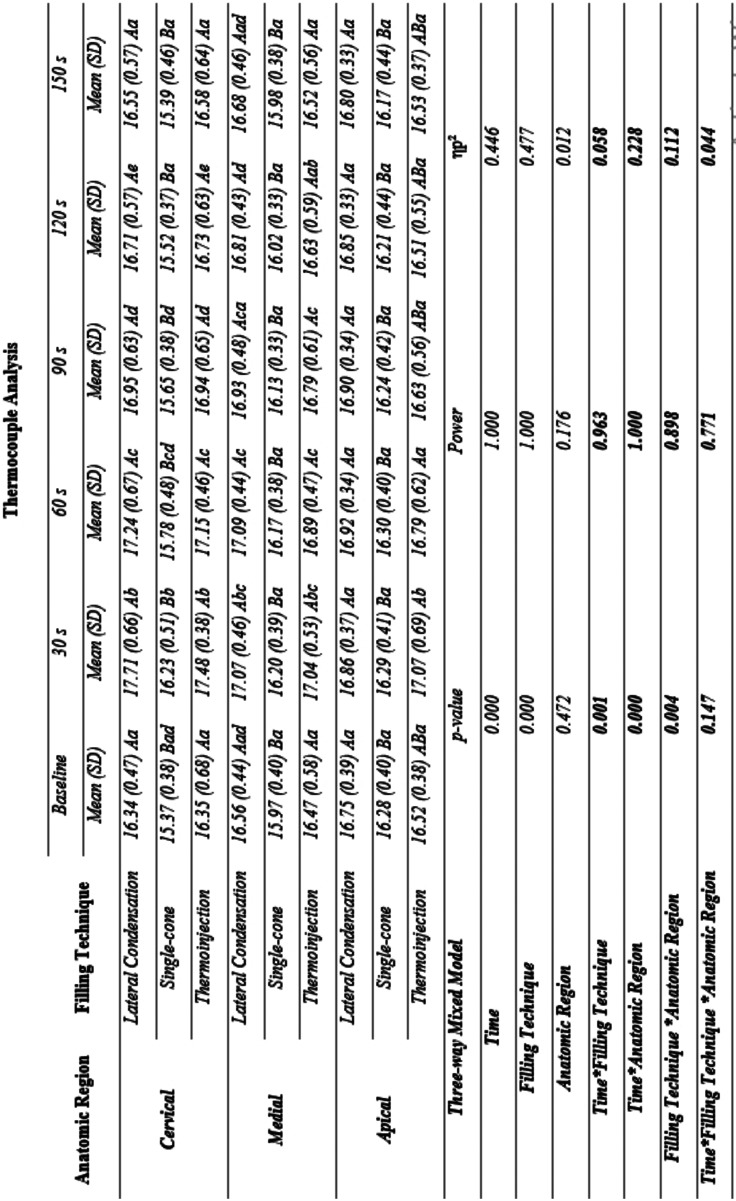
Interaction between time, filling technique, and anatomic region in thermocouple analysis: a Three-way Mixed Model.

Significant interaction effects between time and filling technique (α = 0.001, β-1 = 0.963, ηp2 = 0.058), time and third (α = 0.000, β-1 = 1.000, ηp2 = 0.228), and filling technique and anatomic region (α = 0.004, β-1 = 0.898, ηp2 = 0.112) were found. From these interactions, the magnitude of effect tells us how strongly these factors are related. Specifically, Bonferroni’s post hoc test in each third demonstrated that there is a significant difference between the Single-cone and the other 2 techniques, which do not differ from each other.

Isolating the time factor in each technique, it is possible to observe that the time does not vary in the Single-cone technique in the Media and Apical thirds, whereas a pronounced increase in temperature is expressed in the Cervical third. A relevant increase in temperature is evidenced in Lateral Condensation and Injectable thermoplasticized gutta-percha technique, especially at 30, 60, 90, and 120 s in the Cervical and Media thirds. More importantly, an increase in temperature in the Apical third is only noted in the Injectable thermoplasticized gutta-percha technique.

The temperature in the Single-cone technique in the Cervical third behaves differently from the Media and Apical thirds. No significant difference is indicated in Lateral Condensation and Injectable thermoplasticized gutta-percha techniques across the thirds.

-Temperature assessed with infrared thermography

**Figure 2:**
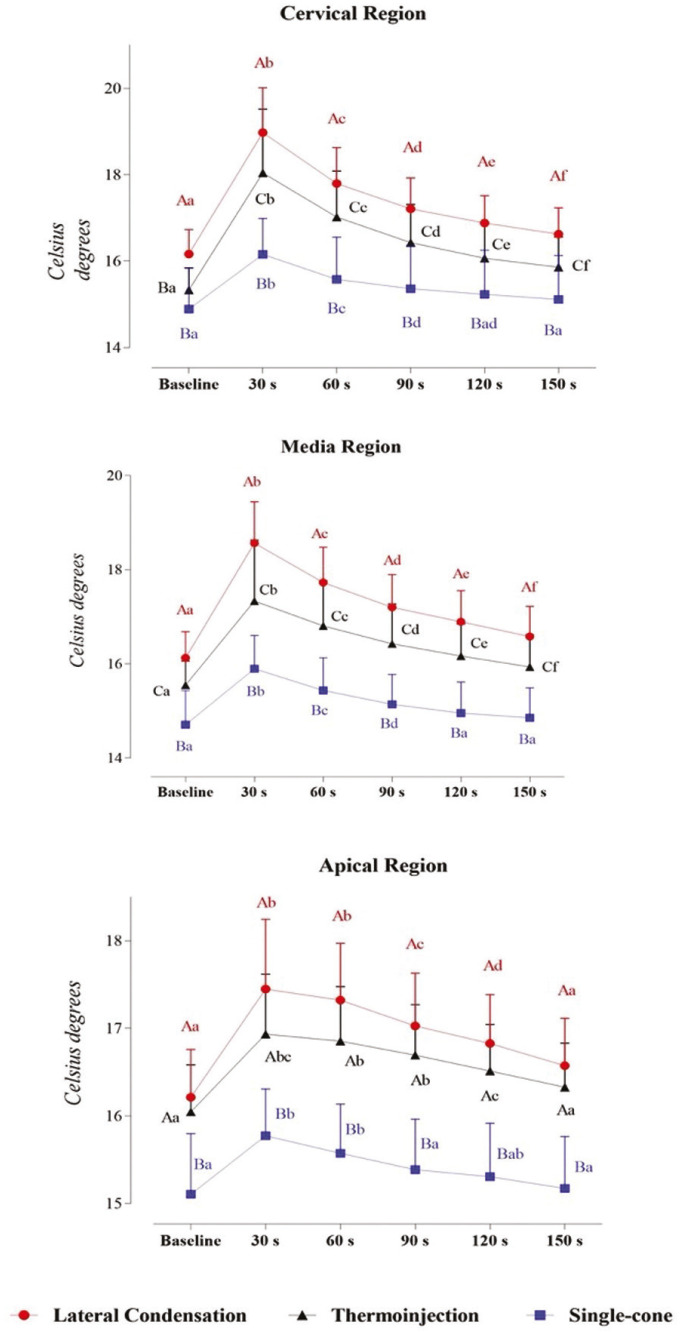
Interaction between time, filling technique, and third in infrared thermographic analysis: a Three-way Mixed Model. Statistical analyses were performed with a sample of 45 cases, 15 per group. ηp2: Partial eta squared. Data plotted as mean and standard deviation. Table 2 provides complementary information regarding data statistics. To overcome sphericity violation, the Greenhouse-Geisser correction were applied. Main effects were suppressed due to significant interactions. Interaction effects for Time and Filling technique: α = 0.001, β-1 = 1.000, ηp2 = 0.174). Interaction effects for Time and Anatomic region: α = 0.000, β-1 = 1.000, ηp2 = 0.258.

Figure 2 shows the three-way mixed model ANOVA and Bonferroni test for multiple comparisons. Table 2 provides complementary information regarding data statistics.

**Table 2:**
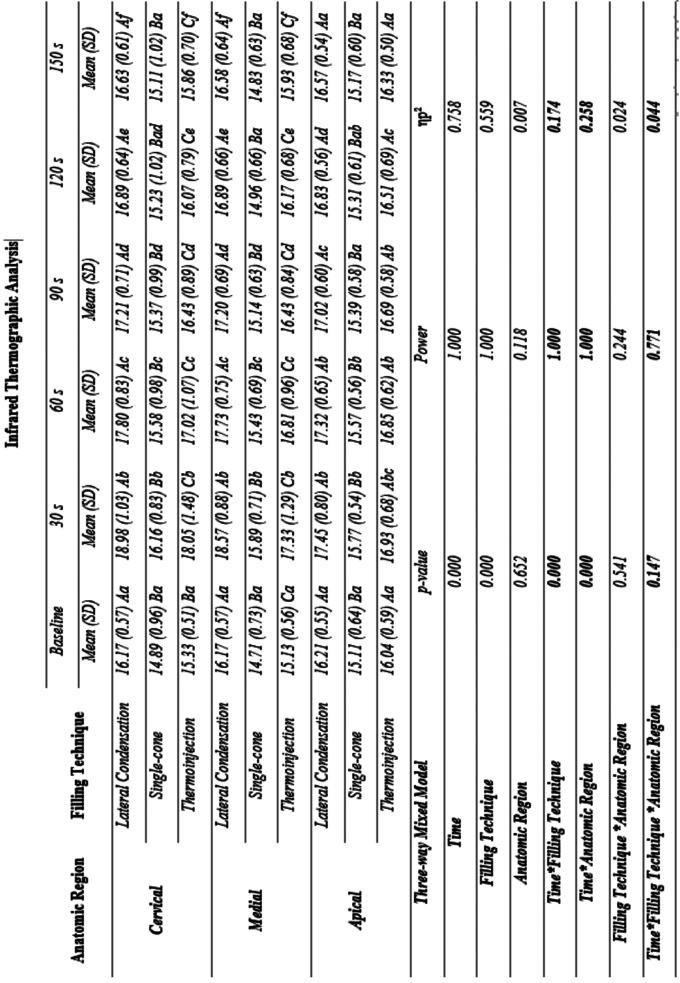
Interaction between time, filling technique, and anatomic region in infrared thermographic analysis: a Three-way Mixed Model.

The third has no significant effect on this ANOVA model due to nonsignificant main and interaction effects. There are significant and strong interaction effects between time and filling technique (α = 0.001, β-1 = 1.000, ηp2 = 0.174) and time and third (α = 0.000, β-1 = 1.000, ηp2 = 0.258). Particularly, multiple matrices comparisons evidence that there is a significant difference between the 3 techniques in the Cervical and Media regions.

In Lateral Condensation and Injectable thermoplasticized gutta-percha technique, it is evidenced an increase in temperature at 30 s followed by a slow decline until 150 s, when it is close to, but still higher than baseline values. Single-cone technique also induces an increase in temperature at 30 s, however, the decline until baseline values occurs at 60 s in the Apical third and 90 s in the Cervical and Media thirds.

## Discussion

The aim of root canal obturation is to provide an endodontic treatment with maximum filling within the canal system previously modeled by different instrumentation protocols. A well-filled canal reduces the chances of reinfection, as bacteria cannot organize and recolonize the tooth (14). In the current research, the significant interaction effects between time and filling technique, time and third, filling technique and third were evaluated using two different temperature measurement methods.

In this study, temperature when assessed using thermocouples only increased significantly within different time intervals during the single cone technique at the cervical third, probably because in this obturation technique, there is contact with the gutta percha only in the cervical superficial portion of the root. During lateral condensation, the instrument spends more time in contact with gutta-percha and this prolonged contact helps temperature rise; Therefore, a temperature increases in the cervical and middle thirds. In the injectable thermoplasticized gutta-percha technique, the gutta percha is already plasticized inside the device when inserted inside the canal, which explains this remarkable temperature difference in the three thirds. Despite that, in all techniques, the temperature did not increase over the 10°C critical limit, in accordance with previous studies (15,16).

When assessing temperature using infrared thermography, significant changes were observed between time and obturation technique and between time and third. This difference was seen in all three assessed techniques, especially in the cervical and middle thirds. It was initially seen an increase in temperature after obturation, followed by a gradual decline, with values close to the initial temperature, but still slightly higher. The fast temperature normalization in the single-cone technique can be explained by the fact that in this technique the gutta percha does not stay in contact with the heated instrument for a long time. Another point to be considered is that Gutta-percha is a poor thermal conductor, transmits heat irregularly, and should be heated 1– 2 mm from the target area, what justifies lower temperatures in the apical third of the root (17).

Previous studies have assessed temperature increase during obturation techniques using thermal cameras (18-20) finding that despite the differences in camera model, different obturation techniques, lack of standardized thermal protocols and low-resolution infrared thermography cameras, that the increase in temperature during root filling with gutta-percha is moderate and safe. Lipski (18) found and acceptable increase in temperature in the continuous wave technique in the upper central incisors; however, the temperature increase exceeded the critical level in the lower central incisors, what indicates that teeth with lower remaining dentin volume and its surrounding tissues may suffer more consequences of the temperature increase during root canal gutta-percha filling. According to Suassuna *et al*. (20), when assessing temperature increase during lateral condensation, single cone and thermomechanical compaction using a thermal camera found that temperature increase generated by these filling techniques are considered within the acceptable range; However, the cervical third needs extra attention to avoid temperature increases over 10°C. Other results also showed that the root temperature increases during obturation in the three techniques, but with small variations (15,16,21).

Thermocouples did not distinguish as much temperature variations as the infrared camera did in this study, because thermocouples have sensors connected to the external portion of the root and detects increases in only three distinct points, unlike the thermographic camera, that evaluates in real time the root temperature during obturation, what within its methodological limitations would be more like the conditions found in a clinical situation.

Considering the methods evaluated, both have their limitations. The thermocouple records the external root temperature at specific points, previously demarcated, in a very isolated way. The thermographic camera, on the other hand, evaluates the temperature of the root, but it cannot record this behavior on video, being necessary to use pre-defined points of the regions of interest, marked in the camera program itself, so that the information could be recorded by an external video of the device’s display.

It is also known that the endodontic filling technique execution time must be taken in consideration when assessing root temperature increase, as the longer the tissues are exposed to heat, the higher the possibility of tissue damage. Among the techniques, the single-cone technique was the method in which heat was applied to the root for the shortest time interval during the obturation technique, therefore, root temperature decreases to values close to the base value in a short time.

Another limitation of this study is that no periodontal support tissues and no local blood circulation were simulated around the root, which would influence the temperature behavior and dissipation. Studies like Zhou *et al*. (22) and Cen *et al*. (23), through a finite element analysis model, considered this variable in their research but also checked small temperature variations.

However, this limitation is inked to tests performed at a laboratory level. To fully understand this issue, patients would need to have their temperature recorded in real time during the filling, a condition that is difficult to replicate, in addition to variations linked to the patient’s phenotype, such as thickness of mucosa, bone thickness and root volume.

A controlled trial would better evaluate the patient’s comfort in the postoperative period and variables such as tissue repair, periodontal health, and tooth survival; however, this methodology is hard to reproduce and would involve new methods of temperature assessment or an intraoral infrared thermal camera, not yet available on the market.

## Conclusions

All assessed obturation techniques increase the temperature of the root, especially the thermoinjection technique. However, temperature increase is not enough and does not last enough to be considered harmful for the surrounding tissues. It is suggested that the triad temperature x obturation technique x tissue reaction should be evaluated by controlled clinical research. The assessment of other variables such as kinematics of instrumentation, internal anatomy and stage of pulp disease can influence temperature increase. Furthermore, it can be concluded that infrared thermography and thermocouples can be used to assess root temperature variation.
